# Differentiation Potential of Early- and Late-Passage Adipose-Derived Mesenchymal Stem Cells Cultured under Hypoxia and Normoxia

**DOI:** 10.1155/2020/8898221

**Published:** 2020-09-18

**Authors:** Ashley G. Zhao, Kiran Shah, Julien Freitag, Brett Cromer, Huseyin Sumer

**Affiliations:** ^1^Department of Chemistry and Biotechnology, Faculty of Science, Engineering and Technology, Swinburne University of Technology, John St, Hawthorn VIC 3122, Australia; ^2^Magellan Stem Cells P/L, 116-118 Thames St, Box Hill VIC 3129, Australia; ^3^Melbourne Stem Cell Centre, Box Hill, VIC 3129, Australia; ^4^Charles Sturt University, School of Biomedical Science, Albury, NSW 2640, Australia

## Abstract

With an increasing focus on the large-scale expansion of mesenchymal stem cells (MSCs) required for clinical applications for the treatment of joint and bone diseases such as osteoarthritis, the optimisation of conditions for *in vitro* MSC expansion requires careful consideration to maintain native MSC characteristics. Physiological parameters such as oxygen concentration, media constituents, and passage numbers influence the properties of MSCs and may have major impact on their therapeutic potential. Cells grown under hypoxic conditions have been widely documented in clinical use. Culturing MSCs on large scale requires bioreactor culture; however, it is challenging to maintain low oxygen and other physiological parameters over several passages in large bioreactor vessels. The necessity to scale up the production of cells *in vitro* under normoxia may affect important attributes of MSCs. For these reasons, our study investigated the effects of normoxic and hypoxic culture condition on early- and late-passage adipose-derived MSCs. We examined effect of each condition on the expression of key stem cell marker genes POU5F1, NANOG, and KLF4, as well as differentiation genes RUNX2, COL1A1, SOX9, COL2A1, and PPARG. We found that expression levels of stem cell marker genes and osteogenic and chondrogenic genes were higher in normoxia compared to hypoxia. Furthermore, expression of these genes reduced with passage number, with the exception of *PPARG*, an adipose differentiation marker, possibly due to the adipose origin of the MSCs. We confirmed by flow cytometry the presence of cell surface markers CD105, CD73, and CD90 and lack of expression of CD45, CD34, CD14, and CD19 across all conditions. Furthermore, *in vitro* differentiation confirmed that both early- and late-passage adipose-derived MSCs grown in hypoxia or normoxia could differentiate into chondrogenic and osteogenic cell types. Our results demonstrate that the minimal standard criteria to define MSCs as suitable for laboratory-based and preclinical studies can be maintained in early- or late-passage MSCs cultured in hypoxia or normoxia. Therefore, any of these culture conditions could be used when scaling up MSCs in bioreactors for allogeneic clinical applications or tissue engineering for the treatment of joint and bone diseases such as osteoarthritis.

## 1. Introduction

Mesenchymal stem cells (MSCs) are multipotent cells, originally derived from the embryonic mesenchyme, and able to differentiate into connective tissues such as bone, fat, cartilage, tendon, and muscle [1, 2]. These cells are ubiquitous and reside in various tissues and organs for self-repair and tissue homeostasis [3]. They can be isolated from bone marrow, periosteum, trabecular bone, adipose tissue, synovium, skeletal tissue, blood, brain, spleen, liver, kidney, lung, bone marrow, muscle, thymus, pancreas, blood vessels, and deciduous teeth [4, 5]. MSCs can self-renew, have immunosuppressive properties, and intrinsically secrete a wide range of bioactive molecules [6, 7]. MSCs have significant clinical value and have been used in cardiovascular, neural, and orthopaedic therapeutic applications such as osteoarthritis. To date, there are 1,052 clinical trials registered for various medical conditions exploring the therapeutic benefits of MSCs in a broad range of diseases (http://clinicaltrials.gov). Furthermore, MSCs derived from adipose tissue show great promise for the treatment of degenerative diseases such as osteoarthritis [8, 9]. Collectively, this activity demonstrates the therapeutic potential of MSCs, widely acknowledged by researchers worldwide.

Human MSCs are heterogeneous and can be obtained from many sources via different isolation, culture, and expansion methods. There are also a variety of different approaches to characterise these cells [10]. This has caused some difficulty in comparing study outcomes and has led to controversial results. Consequently, the Mesenchymal Stem Cell Committee of the International Society for Cellular Therapy (ISCT) has provided three minimal standard criteria to define MSCs for laboratory-based investigation and preclinical studies, based on adherent properties, self-renewal, expression of surface markers, and multilineage differentiation capacity [10]. Firstly, MSCs must be plastic-adherent in tissue culture flasks. Secondly, more than 95% of MSC population must express CD105, CD73, and CD90 and lack expression (less than 2% population) of CD45, CD34, CD14 or CD11b, CD79a or CD19, and HLA class II. Third, MSCs must be able to differentiate into osteoblasts, adipocytes, and chondroblasts *in vitro* with standard differentiation conditions.

MSCs are functionally heterogeneous and often present in limited numbers in the human body [1, 11]. Their *in vitro* expansion for clinical dosage has become a necessity and warrants large-scale production of MSCs prior to implantation. The proliferative properties of MSCs are robust but the lack of standard methods for isolation, the different sources of MSC, and variation in both culture conditions and the number of passages may result in less than optimal cells for clinical purposes. The impact of *in vitro* culture conditions on cellular attributes of MSCs is an important factor to consider for cell therapy. Several studies have described changes in the biology of the cells, including physiological and genetic changes caused by varying tissue *ex vivo* cell culture parameters such as seeding density, media nutrients, length of culture, shear force when culturing in bioreactors, pH, temperature, and oxygen percentage [12–14]. Culture conditions can have an impact on gene expression, the proteome and cellular organization [15, 16]. All these physicochemical parameters are important and careful cell culture optimisation, with these parameters in mind, must be performed in order to produce optimal cells for therapy that have close functional similarity to native stem cells *in vivo*.

Of these parameters, oxygen level in cell culture has been described in the literature as having a significant influence on MSC characteristics. Oxygen tension acts both as a metabolic substrate and a powerful signalling molecule to regulate the proliferation and differentiation properties of stem cells [17]. Quiescent MSCs in their natural niches are tightly controlled and maintained to protect them from oxidative damage at a physiological low oxygen tension [18] and mainly rely on anaerobic glycolysis to support ATP production [19]. However, MSC expansion is often conducted in normoxic conditions (21% O_2_), which is about 4-10 folds greater than their natural physiological environment [20, 21]. Cultured MSCs under high oxygen conditions or normoxia would switch from anaerobic glycolysis to the mitochondrial oxidative phosphorylation which might be harmful for cellular function [22]. Differential culturing of MSCs under hypoxia and normoxia does not seem to affect immune-phenotypic features or cellular plasticity, but does seem to affect cell morphology and complexity, as well as mitochondrial activity [23]. Culturing MSCs at a larger scale may require a bioreactor and increased passaging, however, and it may be challenging to maintain low oxygen and other physiological parameters over several passages. It is unclear what the effects of oxygen concentration are on stem cell marker expression and multipotency.

Hypoxia is one of the key parameters described to exert effects on several cellular activities in MSCs during osteogenic and chondrogenic differentiation [17]. During chondrogenesis, low oxygen tension (5%) inhibits the proliferation of MSCs but increases the total collagen, protein, and glycosaminoglycan synthesis [24]. Other studies support this reduced proliferation rate of MSCs in hypoxic conditions, as well as showing reduced adipogenic and osteogenic differentiation potentials [25]. Nevertheless, there was no significant difference between normoxia (21% oxygen) and hypoxia (2% oxygen) in the cell surface expression of the markers CD73, CD90, CD105, CD106, CD146, and MHC class I, which is measured by flow cytometry. As mammalian tissue has much lower oxygen concentration than atmospheric conditions, ranging from 1 to 7% in cartilage, bone marrow, and 10-13% in arteries lungs and liver [26], low oxygen culture is mostly used *in vitro*. It is believed that low oxygen is able to maintain normal cellular functions such as cell growth, differentiation, and cell migration [26–29].

Nevertheless, the impact of oxygen tension on cultured MSCs remains controversial, due to conflicting results, although these discrepancies might be due to other differences in culture conditions or different sources of MSCs. Therefore, further comparative analyses on *in vitro* cultured cells under normoxic and hypoxic culture conditions are needed to determine the effects on MSC stemness, particularly for large-scale systems such as bioreactors for clinical use. Although bone marrow-derived MSCs were the first identified and have been extensively studied [1], harvesting from bone marrow is a limiting factor as it is a painful procedure and produces only a low yield of MSCs. An alternative source of MSCs from the adipose tissue can be obtained by a minimally invasive procedure and can achieve a 100 to 500-folds greater yield than from the bone. It is now an accepted alternative source of MSCs, leading to changes in medical practices and regenerative medicine [30, 31]. The current study is aimed at comparing the characteristics of early- and late-passage adipose-derived MSCs, which is used for the treatment of osteoarthritis and cultured under hypoxic or normoxic conditions. From this comparison, we aimed to determine whether late-passage MSCs grown in normoxic culture conditions could be a reliable, safe, and effective source of cells for regenerative medicine and tissue engineering for applications such as osteoarthritis. We determined whether early- and late-passage MSCs cultured in hypoxia or normoxia maintain the minimal standard criteria for MSCs, set by the ISCT, for laboratory-based investigation and preclinical studies.

## 2. Material and Methods

### 2.1. Cell Culture

Adipose tissue was harvested by liposuction by a qualified clinician with ethics approval and written consent (Monash University Human Research Ethics Committee number CF 14/2230 2014001175 and registered clinical trial Trial registration: Australian New Zealand Clinical Trials Registry - ACTRN12617000638336.). The adipose tissue was then processed in a clean room facility. In brief, the minced adipose tissue was digested with collagenase to release the stromal vascular fraction (SVF) from mature adipocytes. The stromal vascular fraction (SVF) was cultured in the flask as passage 0. The plastic-adherent adipose-derived MSCs were harvested away from floating SVF cells. MSCs were cultured in basal medium with 2 mM glutamine with 5% FBS (Invitrogen) and grown under hypoxic (2% O_2_) or normoxic (~21% O_2_) conditions for up to nine passages. Subculturing by trypsinisation was performed when MSCs reached approximately 80% confluence. The four sample groups were MSCs cultured in hypoxic conditions, harvested at passage 5 (P5H) or at passage 9 (P9H), or cultured in normoxic conditions, harvested at passage 5 (P5N) or passage 9 (P9N).

MSCs were differentiated into chondrocytes or osteocytes by culturing in Chondrocyte Differentiation Reagent ATCC® PCS­500­051™ for 19 days, or Osteocyte Differentiation Reagent ATCC® PCS­500­052™ for 21 days, respectively. In brief, samples were cultured in 6-well culture plates with a half media change every 3-4 days. Osteogenic differentiation was confirmed by Alizarin Red S staining. Cells were fixed with 4% formaldehyde for 30 mins and then rinsed twice with distilled water, stained with Alizarin Red S solution for 3 minutes, and rinsed three times in distilled water before imaging. Chondrogenic differentiation was confirmed by Alcian Blue staining. Cells were fixed with 4% formaldehyde for 30 mins and then rinsed once with DPBS, stained with Alcian Blue solution prepared in 0.1 N HCl for 30 minutes, and then rinsed three times with 0.1 N HCl. Then, 2 ml of distilled water was added into each well to neutralize the samples before imaging.

### 2.2. Quantitative Real-Time PCR and Statistical Analysis

Total RNA was extracted from cell samples, using a RNeasy Mini Kit (Cat#74104, Qiagen), according to the manufacturer's instructions. The concentration and quality of the extracted RNA were measured using a NanoDrop2000 Spectrophotometer (Thermo Scientific). 5 *μ*g of RNA from each sample was transcribed into cDNA using Tetro cDNA Synthesis Kit (Bioline) following manufacturer's instructions. Primers used for gene expression analysis, listed in Table 1, have been published previously [32].

qRT-PCR was performed by using SsoAdvancedTM Universal SYBR Green Supermix kit (ThermoFisher Scientific). Three independent experiments were performed in triplicate, with GAPDH as the reference gene. The reaction mix preparation and thermal cycling protocol were followed according to SsoAdvancedTM Universal SYBR Green Supermix kit. A Bio-Rad CFX96TM system was used for thermal cycling, with initial denaturing at 95°C for 30 sec, then 40 cycles of denaturing at 95°C for 10 sec, annealing and extension at 59°C for 30 sec, and Melt-Curve Analysis from 65°C to 95°C with 0.5°C increment. Statistical analysis was performed by one-way ANOVA; the P5H samples were set to a value of 1 and used as a reference to determine a statistical significance.

### 2.3. Flow Cytometry

In brief, 0.5 × 10^6^ MSCs were resuspended in 500 *μ*L of FACS buffer (1% BSA and 0.1% EDTA in phosphate-buffered saline (PBS)) and incubated for 30 minutes at 4°C with 1 : 500 dilution of antibodies, CD73 (Cat# 11073942), CD90 (Cat# 25090942), CD105 (Cat# 12105742), or CD14 (Cat# 11014942), CD19 (Cat# 25019382), CD34 (Cat# 25034942), and CD45 (Cat# MHCD4531) from Thermofisher Scientific, respectively. After washing twice with 1 mL of FACS buffer, the labelled MSCs were resuspended in 500 *μ*L of FACS buffer and subjected to flow cytometry (Attune NxT, Life Technologies) to analyse surface markers.

## 3. Results

To determine the effect of oxygen concentration during culture on MSC properties, we cultured MSCs under different oxygen conditions for different numbers of passages. Initially, primary adipose-derived MSCs were cultured under GMP conditions under hypoxia (2% O_2_) up to passage 4. The plastic-adherent cells were subjected to flow cytometry analysis for the CD markers CD73, CD90, CD105, CD14, CD19, CD34, and CD45, to confirm their MSC phenotype before cryopreservation. The cells were then further subcultured under hypoxic or normoxic conditions and harvested at either passage 5 or 9 and frozen for later analysis. This gave rise to four sample groups that were further analysed: MSCs cultured in hypoxic conditions and harvested at passage 5 (P5H) or at passage 9 (P9H), or cultured in normoxic conditions and harvested at passage 5 (P5N) or at passage 9 (P9N).

RNA samples from the four sample groups under normoxia and hypoxia conditions at early and late were analysed by qRT-PCR. Genes were organised into four groups—pluripotent genes, osteogenic genes, chondrogenic, and adipogenic genes. The relative quantification 2^-∆∆CT^ method was used to calculate the relative amount of mRNA templates in each of the test samples from (C_T_ (target, test), C_T_ (target, calibrator), C_T_ (GAPDH, test), C_T_ (GAPDH, calibrator)) four C_T_ values in triplicate [33]. The housekeeping gene *GAPDH* was employed as the reference gene and the low passage sample under hypoxia, P5H, served as the calibrator. The target gene expression in all other samples is thus represented as an increase or decrease relative to the calibrator. Since cDNAs were synthesised from RNAs by one cycle of PCR amplification, the gene expression analysis results represent the relative amount of mRNA templates in the test sample. The errors were calculated from the standard deviations carried by the triplicate C_T_ values using the standard propagation of the error methods.

**Figure 1:**
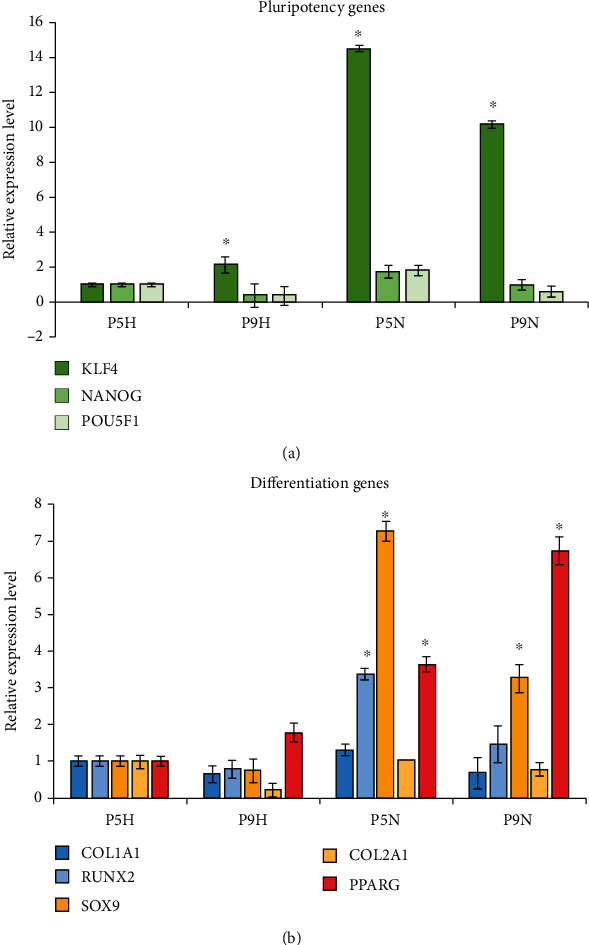
Relative gene expression levels by qRT-PCR for cells cultured under hypoxia and normoxia. (a) qRT-PCR for pluripotency genes *KLF4*, *NANOG*, and *POU5F1.* (b) qRT-PCR for MSC differentiation genes; osteogenic markers *COL1A1* and *RUNX2*, chondrogenic markers *SOX9* and *COL2A1*, and adipogenic marker *PPARG*. Relative expression levels are expressed using the *GAPDH* as the reference gene, and values were normalized to P5H; error bars represent SD. Statistically significant difference, *p* < 0.05, shown by ^∗^ for relative expression level when compared to P5H sample.

The relative gene expression for pluripotent marker genes *KLF4*, *NANOG*, and *POU5F1* (*Oct-4*) are shown in Figure 1. The *KLF4* gene was expressed at markedly higher levels (*p* < 0.01) in MSCs cultured in normoxia than in hypoxia. The levels of *KLF4* increased 2.1-folds with increased passage number from 5 to 9 in hypoxia (*p* < 0.05), while it decreased 0.7-folds in normoxia (*p* < 0.05). Likewise, *NANOG* and *POU5F1* (*Oct-4*) genes were expressed slightly higher under normoxia than in hypoxia, but the change was much smaller than for KLF4. Under both hypoxic and normoxic conditions, the expression of *NANOG* and *POU5F1* (*Oct-4*) decreased with the increasing passage number.

We next turned to gene markers of lineage-specific differentiation. An examination of relative expression of osteogenic marker genes *RUNX2* and *COL1A1* revealed little change with passage number under hypoxia (Figure 1). *COL1A1* also had no significant change with passage number under normoxia; however, the *RUNX2* gene was considerably higher in normoxic culture conditions relative to hypoxic conditions and was significantly higher (*p* < 0.05) at the earlier passage. Expression of the chondrogenic marker genes *Sox9* and *COL2A1* was found to remain relatively low in hypoxia, while *Sox9* expression was significantly higher in normoxia when compared to hypoxia (*p* < 0.01) and decreased 0.45-folds with increased passage number. Overall expression of oestogenic and chondrogenic genes reduced with passage number. In contrast, expression of the adipogenic marker gene *PPARG* was increased markedly in normoxia relative to hypoxia (*p* < 0.01). Expression of *PPARG* also increased with passage number in MSCs cultured in both hypoxia and normoxia (*p* < 0.01). Overall, the expression levels of pluripotent, osteogenic, chondrogenic, and adipogenic marker genes were higher in normoxia when compared to equivalent hypoxia condition. The expression levels for genes reduced with passage number, except for PPARG which increased with passage number in cells grown in both hypoxia and normoxia.

**Figure 2:**
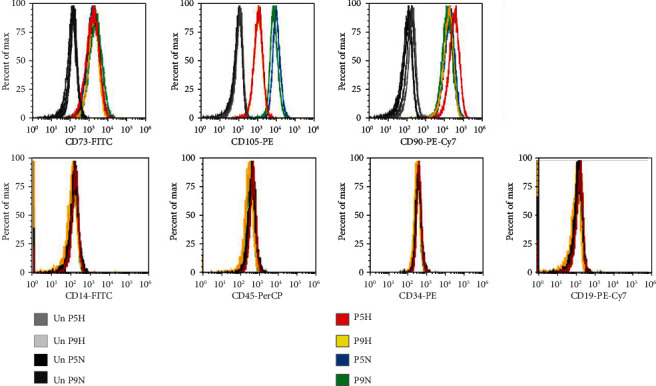
Flow cytometry of CD cell surface markers for cells cultured under hypoxia and normoxia. The positive CD markers for MSCs as detected by the fluorescent antibodies anti-CD73 FITC, anti-CD105 PE, and anti-CD90 PE Cy7. The negative markers of MSCs were detected using anti-CD14 FITC, anti-CD45 PerCP, anti-CD34-R-PE, and anti-CD19 PE-Cy7 antibodies. Unstained cell for each condition was used as negative controls.

Next, we determined the effect of oxygen tension and passage number on MSC cell surface protein markers. High expression of surface markers CD105, CD73, and CD90 are important criteria for the classification and clinical use of MSCs. Flow cytometry was employed to determine the presence of these proteins using the fluorescent antibodies anti-CD105 PE, anti-CD73 FITC, and anti-CD90 PE Cy7. All samples showed high expression of each of the cell surface markers by flow cytometry, relative to unlabelled controls (Figure 2), indicating that the cells retain these positive markers of MSCs at both early and late passages when cultured in either hypoxia and normoxia. Interestingly, the level of CD105 detected on the cell surface was markedly higher for cells grown in normoxia than for those grown in hypoxia. To further confirm MSC state, negative markers of MSCs, CD14, CD45, CD34, and CD 19 were tested by flow cytometry using anti-CD14 FITC, anti-CD45 PerCP, anti-CD34-R-PE, and anti-CD19 PE-Cy7 antibodies, respectively. All cell samples were negative for each of these cell surface markers by flow cytometry, overlapping closely with unstained controls (Figure 2). This further confirms that cells grown under both hypoxia and normoxia, and at early and late passage, meet the standard criteria set by the ISCT to define MSCs for laboratory-based investigation and preclinical studies.

**Figure 3:**
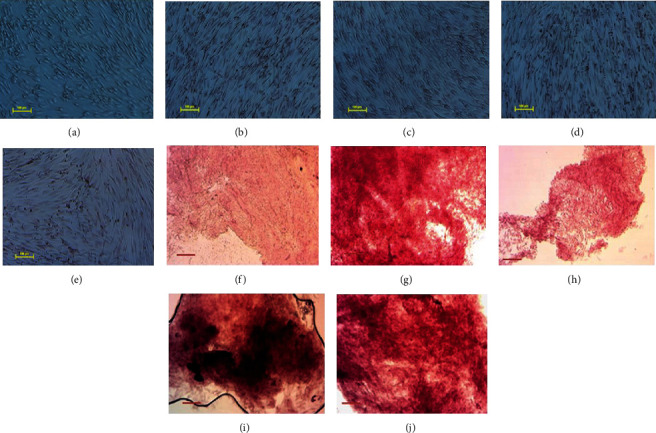
Osteogenic differentiation of MSCs cultured under hypoxia and normoxia. Alizarin Red S staining for cells differentiated for 19 days imaged in (a–e) culture wells and (f–j) on coverslips. (a) Negative control, (b) passage 5 hypoxia (P5H), (c) passage 9 hypoxia (P9H), (d) passage 5 normoxia (P5N), and (e) passage 9 normoxia (P9H). Scale bar 100 *μ*m.

**Figure 4:**
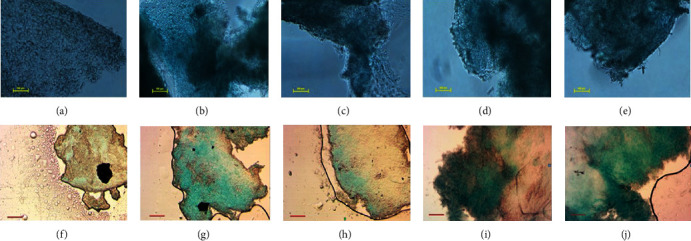
Chondrogenic differentiation of MSCs cultured under hypoxia and normoxia. Alcian Blue staining for cells differentiated for 21 days imaged in (a–e) culture wells and (f–j) on coverslips. (a) Negative control, (b) passage 5 hypoxia (P5H), (c) passage 9 hypoxia (P9H), (d) passage 5 normoxia (P5N), and (e) passage 9 normoxia (P9H). Scale bar 100 *μ*m.

A final characteristic of MSCs is that they can differentiate into multiple lineages under the appropriate conditions *in vivo* and *in vitro*. Thus, we determined the chondrogenic and osteogenic differentiation potential of the adipose-derived MSCs. Osteogenic differentiation was induced for 19 days before staining with Alizarin Red S to test for calcium accumulation. Osteogenic differentiation was confirmed in the culture dishes for all culture conditions, relative to undifferentiated controls, by red staining as well as the beginning of mineral deposits in the form of discrete precipitate foci/nodules (Figures 3(a)–3(e)). This osteogenic differentiation was further analysed under higher magnification on coverslips, where Alzerin Red S staining for calcium accumulation was more evident (Figures 3(f)–3(j)). Although both early- and late-passage MSCs grown under normoxia and hypoxia showed differentiation potential, cells grown under hypoxia appeared to have increased calcium mineralisation (Figures 3(d) and 3(e)). Alternatively, chondrogenic differentiation was induced for 21 days, before being stained with Alcian Blue to show proteoglycan accumulation. Imaging of cell monolayers in culture dishes revealed positive staining of Alcian Blue for all culture conditions compared to controls (Figures 4(a)–4(e)). The chondrogenic differentiation was further analysed under higher magnification on coverslips whereby Alcian Blue staining for chondrocytes and proteoglycans was more evident (Figures 4(f)–4(j)).

## 4. Discussion

MSCs have significant clinical value and have been used in a number of autologous therapeutic applications. MSCs have anti-inflammatory and immunosupressive properties and could also be used in allogeneicTypo transplantation but this would require large numbers of cells [2, 28, 34]. However, for safety purposes, a low *in vitro* passage number of less than 5 is generally used for MSCs in clinical applications [35]. The challenge for allogenic cell therapies, as well as tissue engineering applications, is that low passage MSCs might not yield enough cells for these applications where larger numbers of stem cells are needed. One potential source of allogenic MSCs is from adipose tissue. There are a large number of liposuction surgeries performed every year around the world, where adipose tissue is removed and discarded as medical waste. This excess adipose tissue could serve as a valuable source of MSCs with implied extensive potential for allogenic therapeutics and tissue engineering [16]. In this study, we focused on adipose-derived MSCs which originally reside at low oxygen concentration (<4%) [36] and investigated the effects of oxygen tension and passage number to determine whether they retain their stem cell properties. We compared four samples: passage 5 and 9 MSCs cultured in hypoxic conditions, P5H and P9H, respectively, and passage 5 and 9 MSCs cultured in normoxic condition, P5N and P9N, respectively. These samples were then characterised by qRT-PCR, flow cytometry of CD markers, and differentiation potential.

Firstly, the relative gene expression in each sample was determined using the relative quantification 2^-∆∆CT^ method. The housekeeping gene *GAPDH* was employed as reference gene as it has comparably stable expression [37], and the P5H sample was used as a calibrator. Three pluripotency marker genes, POU class 5 homeobox 1 (*POU5F1*) gene, Nanog homeobox (*Nanog*) gene, and Kruppel-like factor 4 (*KLF4*) genes, were analysed for their expression by qRT-PCR quantification. *POU5F1* gene, also known as *Oct-4*, encodes a transcription factor containing a POU homeodomain involved in embryonic development and stem cell pluripotency [38]. *NANOG* encodes a DNA binding homeobox transcription factor involved in ESC proliferation, renewal, and pluripotency, which can also block stem cell differentiation [39]. *KLF4* gene encodes a Kruppel family transcription factor involved in diverse cellular processes to regulate cell proliferation, differentiation, and acts a suppressor of p53 gene expression [40, 41]. The roles of *Oct-4* and *NANOG* are to maintain MSC properties, keeping MSCs in proliferative and undifferentiated states, while KLF4 regulates the cell cycle. This study has shown that adipose-derived MSCs expressed the classical pluripotency-related genes *NANOG*, *Oct-4*, and *KLF4*. Cells grown in normoxia had a higher expression of the genes than those grown in hypoxia. These results are in line with a study demonstrating that 2,232 genes which involved in development, morphogenesis, cell adhesion, and proliferation were upregulated more than three-folds in MSCs under normoxic culture condition [42]. Additionally, hypoxia has been shown to inhibit the expression of stemness genes in MSCs such as *Oct-4* gene [43]. This is in contrast to other reports whereby hypoxia enhanced stemness gene expression [26, 36, 44–46]. Furthermore, we observed a reduction *NANOG* and *Oct-4* genes' expression with an increased passage number, both in hypoxia and normoxia. This finding corresponds with previous studies in which the expression of two major pluripotent genes *Oct-4* and *NANOG* were expressed at higher level at early passage and reduced with passage number [47, 48]. Additionally, we found that *KLF4* was expressed much higher compared to *NANOG* and *Oct-4,* and was also much higher under normoxia. Furthermore, the increased expression of KLF4 with passage number under hypoxia is in line with previous reports of young versus old human BM-MSCs [49], while the reduction in KLF4 expression under hypoxia versus normoxia has also been previously reported [17]. These results taken together suggest that culturing MSCs under normoxia activates KLF4 to regulate the cell cycle and maintain MSCs in their proliferative and undifferentiated state.

The relative expression for the chondrogenic, osteogenic, and adipogenic genes were also analysed. Firstly, the chondrogenic genes SRY-box 9 (*SOX9*) and Collagen type II alpha 1 chain (*COL2A1*) levels decreased with passage number. The *COL2A1* gene encodes the alpha 1 chain of type II collagen—a fibrillar collagen found in cartilage and *SOX9* is a master regulator of chondrogenesis [50]. These results concur with previous studies, which showed the chondrogenic differentiation potential reduced at higher passage [47, 51, 52]. Next, the adipogenic gene, peroxisome proliferator-activated receptor gamma (*PPARG*) gene, was analysed. It encodes a member of the peroxisome proliferator-activated receptor subfamily of nuclear receptor gamma and is a regulator of adipocyte differentiation. PPARG gene expression increased with passage number for both hypoxia and normoxia, with higher levels detected in normoxia overall. This is inconsistent with previous studies, which have shown that MSCs have a reduced capacity for adipogenic differentiation with increasing passage number [52]. The adipose origin of the MSCs used in this study may be an explanation for this apparent contradiction. In a previous study, bone marrow derived-MSCs differentiated readily into osteoblasts and adipose-derived MSCs into adipocytes [53]. Finally, the osteogenic gene expression for the Runt-related transcription factor 2 (*RUNX2*) and Collagen type I alpha 1 chain (*COL1A1*) genes were also determined. Both genes decreased with passage number, consistent with previous published results [48, 52].

The presence or absence of CD cell surface markers is also important criteria for MSCs. We determined the levels of both positive and negative MSC markers by flow cytometry and qRT-PCR. As a minimum, it has been suggested that at least two positive and two negative markers are required for MSC phenotyping [54]. We found that the three positive CD markers CD105, CD73, and CD90 were present at high levels in all samples, as determined by flow cytometry. Interestingly, CD105 was higher in normoxia when compared to cells cultured under hypoxia; the reduced levels of MSC surface marker expression of CD105 and CD44 have also been reported for MSC cultured in hypoxia for greater than 48 hrs [55]. These studies concluded that the consequences of the downregulation of CD105, which is an adhesive molecule and part of the TGF*β* receptor complex, remain to be determined. Additionally, our findings for the negative MSC CD markers CD14, CD45, CD34, and CD19 were confirmed to be absent across all conditions. Taken together, our results indicate that adipose-derived MSCs grown in both hypoxia and normoxia and at early and late passage meet the minimum CD phenotype requirements for labatory-based investigation and clinical applications [35].

MSCs also have the ability to differentiate into many different cell types. Here, we demonstrated the ability of adipose-derived MSCs to differentiate into osteocytes and chondrocytes under all culture conditions. Firstly, following 19 days of osteogenic differentiation MSCs grown under hypoxia and normoxia were shown to be able differentiate into osteocytes. Positive Alizarin Red S staining and calcium accumulation were detected in both early- and late-passage cells and is in line with the previous results [56, 57]. Additionally, chondrogenic differentiation was induced for 21 days, before being stained with Alcian Blue to confirm MSC differentiation into chondrocytes [58, 59]. Proteoglycan accumulation and deposits were observed for cells cultured in all conditions, confirming chondrogenic differentiation potential of both early- and late-passage MSCs cultured under hypoxia or normoxia.

Although the effects of hypoxia on MSCs have been well studied, there are conflicting reports on its effect on differentiation. For example, hypoxia inhibits osteogenic and adipogenic differentiation capacity of MSCs [25, 60–64] and attenuates MSC chondrogenesis [65]. In contrast, others have shown that hypoxia promotes osteogenic, adipogenic, and chondrogenic differentiation potential of MSCs [26, 66, 67]. Furthermore, hypoxia enhances osteogenesis but inhibits adipogenesis of MSCs [68]. The variation in reports, especially for adipose-derived MSCs, might be caused by different cultivation condition as hypoxia has been shown to effect these cells more [21]. Furthermore, it is important to take into account the normal physiological state [69, 70]. The focus of our study was to determine whether late-passage MSCs grown in normoxic culture conditions could be a reliable, safe, and effective source of cells for regenerative medicine and tissue engineering for applications such as osteoarthritis. We confirmed that late-passage adipose-derived MSCs cultured under normoxia retained both chondrogenic and osteogenic differentiation potential.

In summary, we compared the effects of culturing adipose-derived MSCs in hypoxia and normoxia. We found that the expression levels of pluripotent, osteogenic, chondrogenic, and adipogenic genes were higher in normoxia when compared to hypoxia, and expression levels reduced with passage number. Despite these gene expression changes, we showed that cells grown under all conditions met the phenotypic requirements for both positive and negative CD markers by flow cytometry. Furthermore, the MSCs were confirmed to maintain the ability to differentiate into both osteogenic and chondrogenic cell types. Our findings demonstrate that cells grown under both hypoxia and normoxia, and at early and late passage, meet the standard criteria set by the ISCT to define MSCs for laboratory-based investigation and preclinical studies. Therefore, these culture conditions could be used when scaling up MSC cell culture in bioreactors, if large numbers of cells are required for allogeneic clinical applications or tissue engineering for the treatment of joint and bone diseases such as osteoarthritis.

## Figures and Tables

**Table 1:** The information of the specific genes.

Gene marker type	Name of gene primer	Gene name	Accession number	Forward and reverse sequences	PCR product length (bps)
Housekeeping	Glyceraldehyde-3-phosphate dehydrogenase	*GAPDH*	NM_002046.5	F: ATGTTCGTCATGGGTGTGAA R: TGTGGTCATGAGTCCTTCCA	144
Pluripotent gene	POU class 5 homeobox	*POU5F1*	NM_002701.5	F: GCAATTTGCCAAGCTCCTGAA R: AGCTAAGCTGCAGAGCCTCAAAG	141
	Nanog homeobox	*NONAG*	NM_024865.3	F: CAACTGGCCGAAGAATAGCAATG R: TGGTTGCTCCAGGTTGAATTGTT	159
	Kruppel-like factor 4	*KLF4*	NM_004235.5	F: AAGAGTTCCCATCTCAAGGCACA R: GGGCGAATTTCCATCCACAG	91
Osteogenic gene	Runt-related transcription factor 2	*RUNX2*	NM_004348.3	F: ATGTGTTTGTTTCAGCAGCA R: TCCCTAAAGTCACTCGGTATGTGTA	195
	Collagen type I alpha 1 chain	*COL1A1*	NM_000088	F: GCTACCCAACTTGCCTTCATG R: TGCAGTGGTAGGTGATGTTCTGA	168
Chondrogenic gene	SRY-box 9	*SOX9*	NM_000346.3	F: TGTATCACTGAGTCATTTGCAGTGT R: AAGGTCTGTCAGTGGGCTGAT	187
	Collagen type II alpha 1 chain	*COL2A1*	NM_001844.4	F: TGAAGGTTTTCTGCAACATGGA R: TTGGGAACGTTTGCTGGATT	67
Adipogenic gene	Peroxisome proliferator-activated receptor gamma	*PPARG*	NM_005037.5	F: TGGAATTAGATGACAGCGACTTGG R: CTGGAGCAGCTTGGCAAACA	182

## Data Availability

No data were used to support this study.
